# Low Vitamin D Levels Do Not Predict Hyperglycemia in Elderly Endurance Athletes (but in Controls)

**DOI:** 10.1371/journal.pone.0157695

**Published:** 2016-06-15

**Authors:** Helmuth Haslacher, Sonja Nistler, Delgerdalai Batmyagmar, Elisabeth Ponocny-Seliger, Thomas Perkmann, Thomas M. Scherzer, Michael Kundi, Georg Endler, Franz Ratzinger, Alexander Pilger, Oswald F. Wagner, Robert Winker

**Affiliations:** 1 Department of Laboratory Medicine, Medical University of Vienna, Vienna, Austria; 2 Health and Prevention Center, Sanatorium Hera, Vienna, Austria; 3 Department of Public Health, Medical University of Vienna, Vienna, Austria; 4 Empirical Research, Vienna, Austria; 5 Gruppenpraxis Labors.at, Vienna, Austria; 6 Division of Occupational Medicine, Medical University of Vienna, Austria; University of Saarland Medical School, GERMANY

## Abstract

**Background and Aim:**

Recent studies revealed a link between hypovitaminosis D_3_ and the risk for hyperglycemia. Further mechanistic and interventional investigations suggested a common reason for both conditions rather than a causal relationship. Exposure to sunlight is the most relevant source of vitamin D_3_ (25(OH)D), whereas adipose tissue is able to store relevant amounts of the lipophilic vitamin. Since running/bicycling leads to increased out-door time and alters physiological response mechanisms, it can be hypothesized that the correlation between hypovitaminosis D_3_ and hyperglycemia might be disturbed in outdoor athletes.

**Methods:**

47 elderly marathoners/bicyclists and 47 age/sex matched controls were studied in a longitudinal setting at baseline and after three years. HbA1c as a surrogate for (pre-)diabetic states was quantified via HPLC, 25(OH)D levels were measured by means of chemiluminescent assays. Physical performance was assessed by ergometry.

**Results:**

When adjusted for seasonal variations, 25(OH)D was significantly higher in athletes than in controls. 25(OH)D levels inversely correlated with triglycerides in both groups, whereas only in controls an association between high BMI or low physical performance with hypovitaminosis D_3_ had been found. Likewise, the presence of hypovitaminosis D_3_ at baseline successfully predicted hyperglycemia at the follow up examinations within the control group (AUC = 0.85, 95% CI [0.74, 0.96], p < .001, statistically independent from BMI), but not in athletes.

**Conclusion:**

Our data suggest that mechanisms of HbA_1c_ elevation might differ between athletes and controls. Thus, intense physical activity must be taken into account as a potential pre-analytic confounder when it is aimed to predict metabolic risk by vitamin D_3_ levels.

## Introduction

During the last decade, an inverse relationship between peripheral 25(OH)D (vitamin D_3_) levels and insulin resistance has been clearly established. However, the underlying mechanisms are not yet fully elucidated and it appears that 25(OH)D deficiency might be rather a result than a cause of metabolic dysregulation [1]. This assumption is supported by recent meta-analyses that could not identify a considerable therapeutic effect of cholecalciferol supplementation on metabolic diseases [2,3]. This does not mean that the correlation between vitamin D and insulin resistance is of no clinical interest. In fact, circulating levels of vitamin D derivates could be very valuable for estimating patients’ individual risks. In this regard, Kabadi et al. reported that the interaction between serum 25(OH)D and body mass index (BMI) accounts for 47% of the increased odds for developing an insulin resistance [4]. Likewise, Clemente-Postigo and coworkers described significantly lower levels of 25(OH)D in prediabetic and diabetic patients when compared to individuals without a pathological glycemic status. This effect did not depend on BMI. [5] Heidari et al. added serum 25(OH)D concentrations to the Framingham Risk Score for prediction of adverse cardiovascular events in patients suffering from type 2 diabetes mellitus (T2D). This modification successfully led to a 29% reclassification rate of the study population. [6]

However, the discriminative power of a biomarker can be influenced by different pre-analytical conditions, including high physical activity. This is not far-fetched, since exercise influences and alters a broad range of physiologic processes [7,8]. Sanchis-Gomar and Lippi [9] depicted in a recent review article various settings, in which physical activity influences laboratory test results. In this regard, the authors mention changes in biomarker concentrations of cellular compounds, hemostasis, inflammation, cell destruction, renal function and iron metabolism as well as in hormone concentrations, which are mainly caused by shifts in plasma volume, elevated basal metabolism and increased cellular damage. 25(OH)D is produced from 7-dehydrocholesterol (provitamin D_3_) via previtamin D_3_. The initial reaction occurs within the skin during exposure to ultraviolet B radiation. [10] Thus, it is obvious that a higher exposure to sunlight due to outdoor activities influences circulating levels of 25(OH)D [11]. Granted that 25(OH)D deficiency is more likely a symptom of nascent metabolic disorders than a trigger [1], outdoor exercise would affect the prognostic abilities of 25(OH)D. We thus aimed to investigate, whether 25(OH)D is able to differentially predict a deterioration in glycemic control when compared between a group of elderly endurance athletes (marathon runners/bicyclists) and a control group matched for age and sex.

## Materials and Methods

### Study Design

In 2009, we prospectively enrolled 56 athletes consisting of marathon runners and endurance bicyclists and a control group composed of 58 participants, which were matched for age and sex. Three years after study inclusion, 49 athletes and 49 control participants were reevaluated in a first follow up examination. For further details on the study design see [12–15].

Inclusion criteria for athletic participants:

1. Participation in ≥ 1 of the following competitions during the previous three years: Wachau Half Marathon (21.5 km), Vienna City Marathon (43 km), Carinthian Marathon (180 km bicycle)
2. ≥ 2 hours physical training/week
3. Age ≥ 60 years

Relevant exclusion criteria:

1. Clinically manifest cardiovascular diseases
2. Chronic alcoholism (> 60g daily intake or diagnosed history of alcohol abusus)
3. Unwillingness to give written informed consent

The investigation protocol as well as associate amendments were reviewed and approved by the local ethics committee of the Medical University of Vienna (assigned reference number: EK 401/2005). All medical procedures conformed to institutional guidelines as well as the Declaration of Helsinki and its further amendments. All participants gave written informed consent prior to study inclusion and follow up assessments.

### Biochemical analyses

Laboratory analyses were conducted from fresh (HbA_1c_, triglycerides) and frozen (Vitamin D_3_, <-70°C) biomaterial. At baseline, HbA_1c_ and triglyceride levels were measured at Labors.at, a Viennese group practice for medical and chemical laboratory diagnostics. In detail, HbA_1c_ was analyzed on a fully automated HPLC system (HA-8160, Menarini Diagnostics, Italy) out of K_3_EDTA-anticoagulated blood according to the manufacturer’s instructions. Triglycerides were determined enzymatically from blood serum on an Abbott Architect c8000 platform (Abbott Laboratories, Illinois, USA). Quantification of baseline 25(OH)D and follow up parameters was performed at the Department of Laboratory Medicine, Medical University of Vienna, which is the central laboratory of the General Hospital of Vienna and operates a certified (ISO 9001:2008) and accredited (ISO 15189:2008) quality management system (http://www.kilm.at). For 25(OH) measurement, frozen blood serum was obtained from the MedUni Wien Biobank, a central facility for sample logistics integrated into the quality management system of the Department of Laboratory Medicine (www.biobank.at), and 25(OH)D was quantified by means of chemiluminescent immunoassays on a LIAISON® (DiaSorin, Saluggia, Italy) using commercially available kits (LIAISON® 25 OH Vitamin D total assay, REF# 310600). HbA_1_c was assessed from fresh EDTA-anticoagulated whole blood by HPLC-based separation of hemoglobin fractions on a VARIANT™ II TURBO (Bio-Rad Laboratories Inc., Hercules, USA) using HbA_1c_ Kit-2.0 (REF# 270-2455EX, Bio-Rad).

### Ergometry

Workload was increased every two minutes by 25 W, beginning with 25 W and going on until the point of exhaustion (Ergometrics 900, ergoline GmbH, Bitz, Germany). The individual physical working capacity was expressed as the individual maximal capacity [W] in percent of a reference value standardized for sex, age and body surface [16].

### Classifications

Diabetes mellitus was defined according to the recommendations of the American Diabetes Association as HbA_1c_ ≥ 6.5% and levels between 5.7% and 6.4% were considered as pre-diabetes [17]. At the 3 years follow up examination, HbA_1c_ levels were reassessed. Individuals that remained hyperglycemic or switched from a normoglycemic to a (pre-) diabetic state were classified as “hyperglycemic”, all other participants as “normoglycemic”. This was considered as the primary study end point.

### Statistical analysis

Data showing a Gaussian distribution are presented as mean and standard deviation, other continuous variables as median and interquartile range. Categorical data are given as counts and percentages. Categorical variables were compared by Pearson’s χ² tests. Differences in distributions of continuous variables were estimated by general linear models, Student’s t tests and Mann-Whitney U tests. Exposure to ultra violet B radiation, which is necessary for cutaneous 25(OH)D synthesis, depends on the angle of the sunlight [18]. Hence, the date of blood sampling (day of the year) was chosen as a covariate in a correlation analyses (except for the correlation between actual training amount and 25(OH)D). Binary logistic regression models were computed to identify independent predictors. Receiver operator characterstics (ROC) curves were drawn in order to assess the models’ quality criteria by interpretation of the area under the curve (AUC). P-values were interpreted two-sided, except for directed hypotheses (one-sided interpretation, indicated by p_one-sided_). To control for multiple testing, *p* values were adjusted according to the Benjamini-Hochberg procedure [19] within each bundle of hypotheses (descriptive data, primary hypothesis, correlations). P-values < 0.05 were considered statistically significant.

All calculations were performed using SPSS 22.0 (IBM, Armonk, USA) and MedCalc Statistical Software version 15.8 (MedCalc Software bvba, Ostend, Belgium). Graphs were drawin with MedCalc 15.8 (MedCalc Software bvba) and GraphPad Prism 6.0 (GraphPad Software Inc., La Jolla, USA).

## Results

136 persons were recruited for study participation. Of those, 27 persons were excluded (a detailed list can be derived from [15]) already at baseline. After three years, 49 control participants and 49 athletes could be re-evaluated at the follow up procedure. Since particular data (HbA_1c_,BMI) was missing for four participants, the final study population consisted of 47 (♀ = 4) elderly marathon runners/bicyclists and 47 (♀ = 5) control participants. Three individuals, all of them controls, reported to take oral antidiabetic medication, a single control participant indicated to take an oral 25(OH)D preparation.

**Fig 1:**
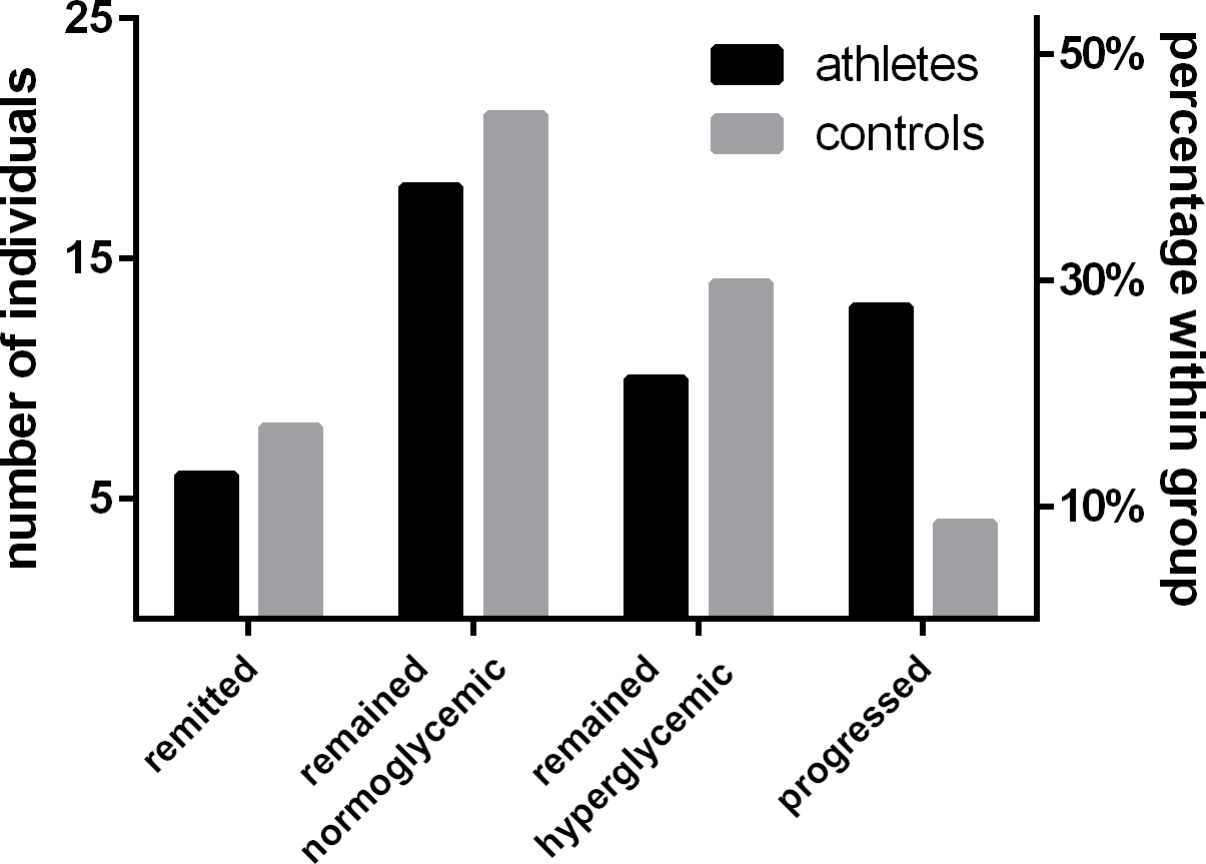
Glykemic state and physical activity. Descriptive depiction of the development of glycaemia during a three years follow up period.

Detailed characteristics of the study population can be retrieved from Table 1. At baseline, serum 25(OH)D levels did not differ significantly between athletes and controls, t(92) = 0.39, p = .969. However, the difference in 25(OH)D concentrations became significant after controlling for seasonal 25(OH)D variations by comparing the estimated marginal means of a general linear model providing the date of blood sampling as a covariate, F(1, 91) = 7.77, p = .018. Moreover, the percentage of baseline glycated HbA_1c_ was modestly lower in athletes, U = -2.94, p = .014. After the 3 years follow up period, 23 athletes (48.9%) and 18 control participants (38.3%) were classified as hyperglycemic. Of those, 13 (27.7%) athletes and 4 controls (8.5%) were reclassified, since they presented normoglycemic at baseline. In contrast, 6 athletes (12.8%) and 8 controls (17%), which were classified as hyperglycemic at baseline, remitted to a normoglycemic state. 18 (38.3%) athletes and 21 (44.7%) controls did not alter their normoglycemic state (Fig 1). Along with these changes, differences in HbA_1c_ between athletes and controls disappeared at follow up, 5.6% (5.4–5.9) vs. 5.5% (5.2–5.9), U = -0.75, p = .683.

**Table 1:** Characterization of the APSOEM study cohort.

		Athletes (n = 47)	Control group (n = 47)	p
Sex		♀ = 4; (8.5%) ♂ = 43 (91.5%)	♀ = 5 (10.6%); ♂ = 42 (89.4%)	.817
Age [years]		65.0 (61.0–68.0)	66.0 (63.0–68.0)	.789
BMI [kg/m²]		23.3 (22.2–25.0)	26.2 (24.6–29.6)	< .001
25(OH)D [nmol/l]^a^		53.4±19.7	53.6±21.2	.969
25(OH)D [nmol/l]^a^^,^ ^c^		61.1±22.9	46.0±22.9	.018*
HbA_1c_ [%]^a^		5.5 (5.3–5.7)	5.6 (5.5–5.9)	.014*
Glycemic status ^a^				.065
	Normoglycemic	31 (66.0%)	25 (53.2%)	
	Prediabetes	16 (34.0%)	16 (34.0%)	
	Diabetes mellitus	0 (0.0%)	6 (12.8%)	
HbA_1c_ [%]^b^		5.6 (5.4–5.9)	5.5 (5.2–5.9)	.683
Glycemic status^b^				.068
	Normoglycemic	24 (66.0%)	29 (53.2%)	
	Prediabetes	22 (34.0%)	12 (34.0%)	
	Diabetes mellitus	1 (0.0%)	6 (12.8%)	
Oral 25(OH)D		0 (0.0%)	1 (2.0%)	
Antidiabetic drugs		0 (0.0%)	3 (6.1%)	

^a^ Baseline

^b^ Follow up

^c^ Comparison of vitamin D concentrations controlled for date of blood sampling (day of the year).

* p < 0.05. P-values were recalculated according to the Benjamini-Hochberg procedure to adjust for multiple testing.

To test our primary hypothesis whether the levels of 25(OH)D are predictive for changes of the glycemic state in outdoor-active athletes and age/sex-matched controls, we performed binary logistic regression models for each cohort. Baseline 25(OH)D, day of blood sampling and BMI were provided as independent variables and hyperglycemia at follow up as outcome variable. For this, 25(OH)D levels were dichotomized into deficient/insufficient (<50 nmol/l) and adequate/optimal (≥50 nmol/l) according to [18]. Within the control group, the model yielded high statistical significance, χ²(3) = 19.76, p < .001. Accordingly, the odds of being hyperglycemic were 5.05 times higher, 95% CI (1.08, 23.63), for individuals with an impaired 25(OH)D status. However, this effect could not be replicated for athletes, since the applied model as well as the odds for 25(OH)D insufficient/deficient participants were statistically insignificant χ²(3) = 2.86, p = .414; OR = 1.03, 95% CI (0.30, 3.58).

**Fig 2:**
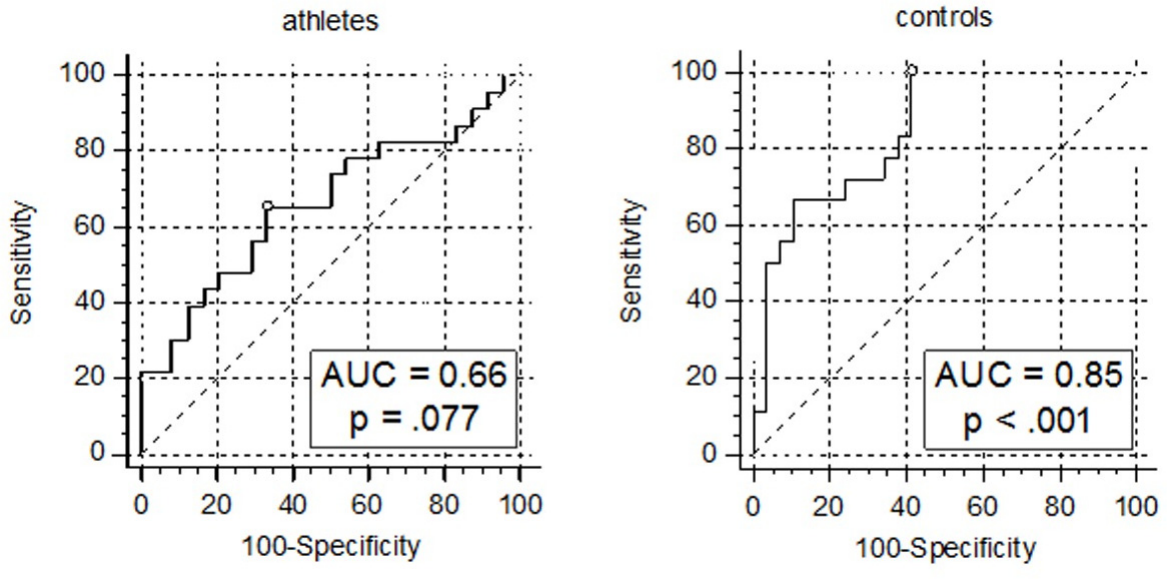
ROC analyses of models including 25(OH)D status for the prediction of future hyperglycemia. Sensitivity and 1-specificity are indicated in %. The blank circles mark the points of the curves corresponding to the Youden’s indices.

Furthermore, AUCs of the models’ ROCs’ were computed in order to evaluate their goodness of fit. Among controls, the AUC of 0.85, 95% CI(0.74, 0.96), p < .001, can be interpreted as indicative of a good discriminative capacity according to [20] (Fig 2). Finding an optimal cut-off point using the Youden’s Index method, the model yielded 100.0% sensitivity and 58.6% specificity, when predicted probabilities were >14%.

As expected, the calculated model for athletes did not show discriminative capabilities better than the toss of a coin, AUC = 0.66, p = .077.

**Fig 3:**
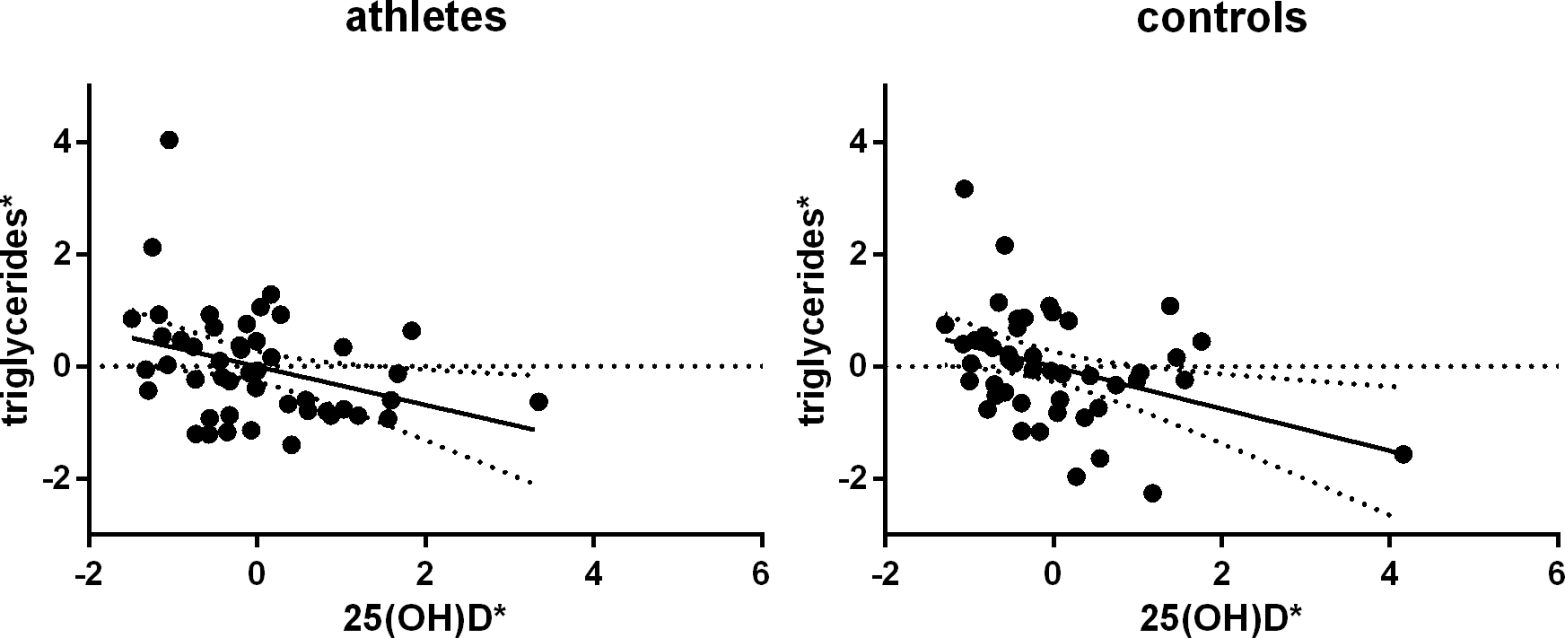
Partial correlation between 25(OH)D and serum triglyceride levels in athletes and controls. *residuals (triglycerides/date of blood withdrawal) are plotted versus residuals (25(OH)D/date of blood withdrawal)

In order to clarify the underlying mechanisms, we tested whether there was a link between hypovitaminosis D_3_ and other metabolic risk factors, as f.e. high serum triglycerides, which was indicated by the literature [21]. Indeed, lower baseline 25(OH)D levels were associated with higher serum triglycerides as calculated by partial correlations in both cohorts: athletes partial r = -0.343, p_one-sided_ = .023; controls partial r = -0.375, p_one-sided_ = .018 (Fig 3).

**Fig 4:**
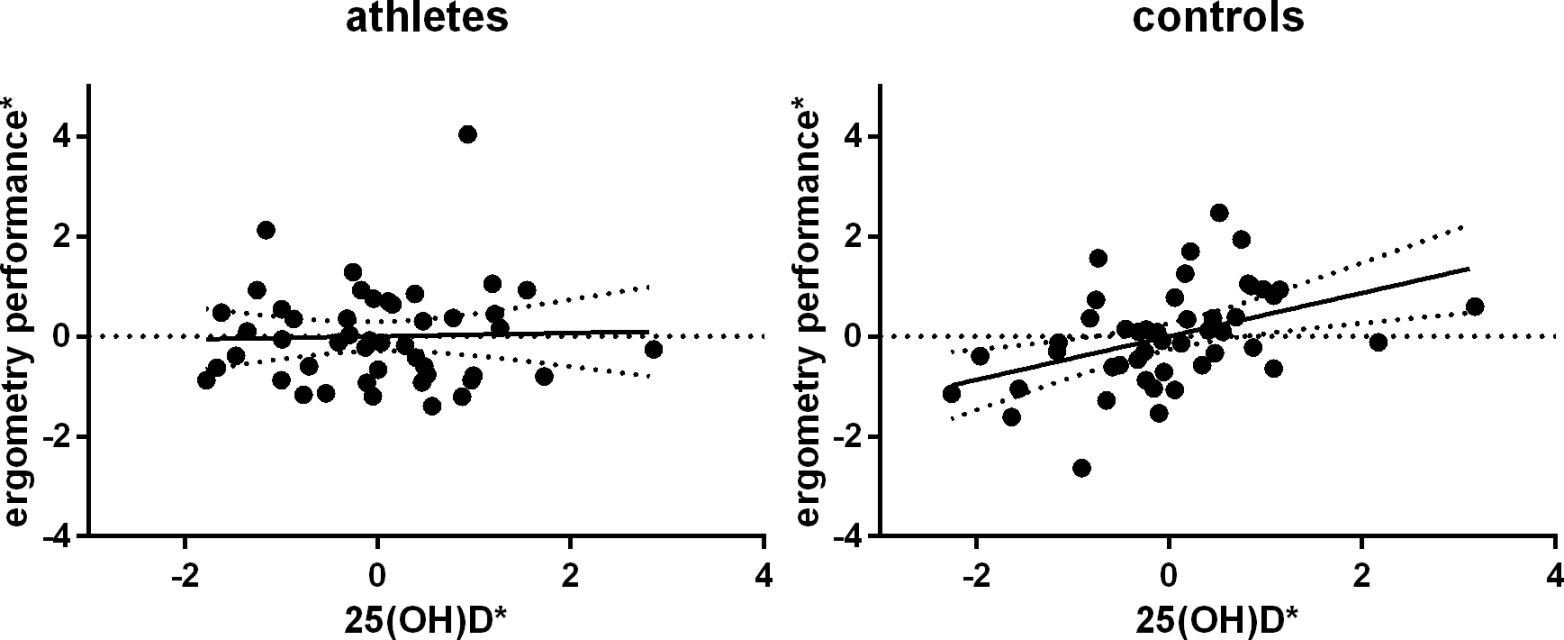
Partial correlation of 25(OH)D and ergometry performance among controls, but not in athletes. *residuals (ergometry performance/date of blood withdrawal) are plotted versus residuals (25(OH)D/date of blood withdrawal)

Subsequently, we aimed to assess if lower 25(OH)D levels are as a consequence associated with a higher BMI. Indeed, there was a weak but significant negative partial correlation in control participants, partial r = -0.282, p_one-sided_ = .041, but not in athletes, partial r = 0.175, p = .142. The same applies to correlations between 25(OH)D concentrations and ergometry performances, which were considered a surrogate for an individual’s physical capacity. Whereas a highly significant positive correlation between the both parameters was found among controls, partial r = 0.434, p_one-sided_ = .007, there was no linear relationship between vitamin D and physical performance in athletes, partial r = 0.033, p_one-sided_ = .414 (Fig 4).

**Fig 5:**
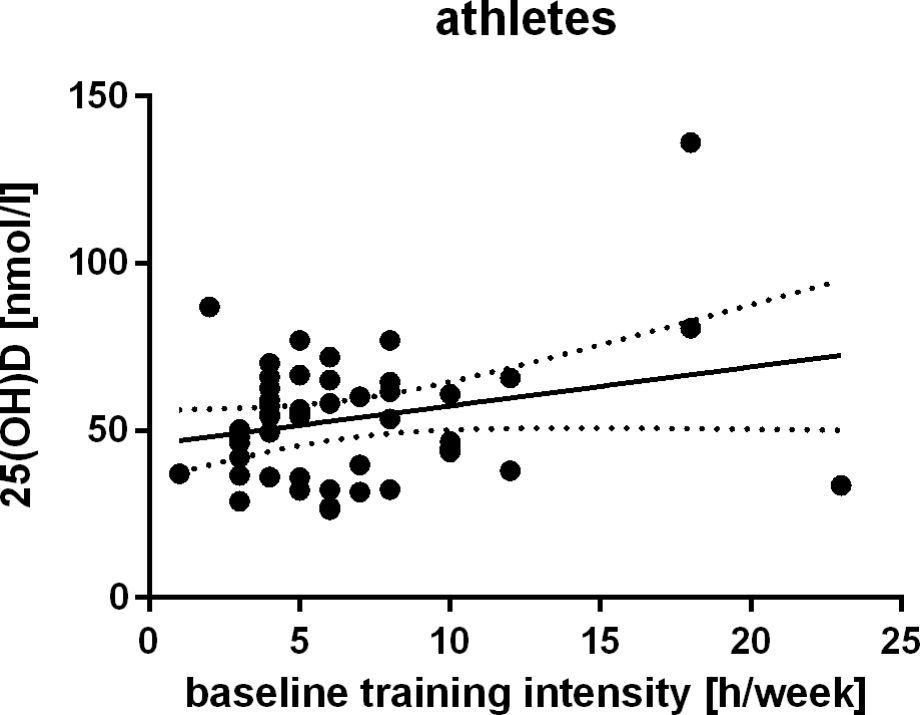
Training intensity and 25(OH)D. Relationship between weekly amount of physical exercise (as a surrogate for outdoor activities) and 25(OH)D levels among athletes.

Athletes reported a median training intensity of 5 hours per week (4–8) at baseline and of 6–7 hours per week (4–10) at the re-examinations three years after, resulting in a median average training amount of 5–6 hours per week within the follow up period (4–9). Marathon training strategies are generally running-based, often with increasing mile goals throughout the training period [22]. As the amount of physical exercise could hence be considered as a surrogate for an athlete’s outdoor activities, we tested whether 25(OH)D levels were positively related to baseline training intensities Indeed, the calculations lead to a significant result: r = 0.259, p_one-sided_ = .039 (Fig 5).

## Discussion

The objective of this study was to determine whether intense physical exercise affects the predictive capacity of vitamin D levels on changes of the glycemic state. This is of particular interest, since clinically manifest insulin resistance is a highly prevalent cause of premature mortality. Currently, worldwide approximately 400 million people are affected by type 2 Diabetes mellitus (T2D). Even more people might be classified as pre-diabetic and are therefore at high risk of developing diabetes. Early identification of those individuals at risk represents a key strategy to combat the disease. [23]

Indeed, our results indicate that the relationship between vitamin D levels and future hyperglycemia might not be present in endurance athletes. However, a combination of 25(OH)D status, BMI and the date of sample withdrawal (to control for seasonal vitamin D variation) helped identifying future (pre-)diabetes with a sensitivity of 100.0% and a specificity of 58.6% among control participants. This observation is in line with existing literature [24]. Notably, there is no strong evidence for a causal relationship between 25(OH)D levels and insulin resistance, since meta-analyses of studies that aimed to decrease the risk for T2D by 25(OH)D supplementation could not calculate any significant impact. George et al., for example, did not find significant effects of 25(OH)D supplementation on fasting blood glucose, Z = 0.54, p = .59, HbA_1c_, Z = 0.24, p = .81, or insulin resistance, Z = 1.04, p = .30 in the total study population. Only those, which had already an impaired glucose tolerance at baseline seemed to benefit from oral vitamin D_3_ to a certain extend (fasting blood glucose: Z = 2.47, p = .01; insulin resistance: Z = 2.22, p = .03). [25] Furthermore, a meta-analysis by Poolsup et al. reported no significant influence of 25(OH)D supplementation regarding the improvement of the HOMA-IR index, Z = 0.49, p = .69, or the 2 hours plasma glucose level after oral glucose tolerance testing (oGTT), Z = 1.07, p = .29. The authors reported only small but significant effects on fasting plasma glucose levels, Z = 2.76, p = .006 and–restricted to patients with prediabetes–in HbA_1c_ proportions, Z = 2.86, p = .008. [2]

Although a clear causal relationship between hypovitaminosis D_3_ and metabolic diseases could not be established and therefore might not be considered as therapeutic option, the reported correlation between 25(OH)D and hyperglycemia could indeed be useful for diagnostic purposes. With this in mind, Song et al. analyzed data from prospective studies investigating the association between baseline 25(OH)D levels and the risk for development of T2D. Their meta-analysis included 21 publications studying a total number of 76,220 individuals. The authors found a relative risk of 0.96, 95% CI(0.94, 0.97), per 10 nmol/L increment in circulating 25(OH)D levels. [26] In a further meta-analysis including 16 cohorts (72,204 participants, 4,877 events), Afzal et al. came to the same conclusion. They reported an odds ratio of 1.50, 95% CI(1.33, 1.66) for developing T2D when comparing the highest versus the lowest 25(OH)D group. [27] This is in-line with our findings among the elderly control participants. The odds for remaining hyperglycemic or switching to a (pre-)diabetic state were 5.05, 95% CI (1.08, 23.63) among those with serum 25(OH)D levels < 50 nmol/l in a model controlling for BMI. Moreover, the model yielded a good AUC, 0.85, p < .001, in ROC-analyses.

Obesity, which is a common comorbidity of (pre-)diabetes, could be a possible explanation for our findings. In detail, we found negative correlations between serum triglycerides and 25(OH)D levels in both, athletes and control participants. Results from Lupton et al, who studied a total of 20,360 adult US inhabitants, support this finding [21]. The authors reported 26.4% higher triglyceride levels in 25(OH)D deficient patients. Again, it is improbable that vitamin D directly affects triglyceride levels. It is more likely that low vitamin D_3_ levels are a result of the high 25(OH)D storage capacity of adipose tissue, as described by Wortsman et al. [28]. In fact, 25(OH)D levels correlated slightly inverse with BMI and positively with ergometry performance in our control group.

In our prospective cohort study, no predictive capability of Vitamin D_3_ levels regarding further hyperglycemia was found among athletes. What could be the reasons for this? A possible explanation might be that 25(OH)D decreased HbA_1c_ solely in patients whose glucose metabolism is already impaired [2] and that athletes might have a better insulin response because of their physical activity. However, this hypothesis cannot be supported by our findings. At baseline, HbA_1c_ concentrations were only slightly lower in athletes, 5.5% (5.3–5.7) vs. 5.6% (5.5–5.9), p = .014. After the three years follow up period, a high proportion of athletes (N = 13, 27.7%) progressed to a hyperglycemic state, whereas HbA_1c_ of only 4 (8.5%) controls rose above 5.7%. As a consequence, the initial small difference between athletes completely disappeared, p = .683. This is not surprising, since it has been shown that intense exercise–as it might be practiced by marathon runners and marathon bicyclists–rather increases plasma glucose levels [29]. As the underlying mechanism, it has been proposed that the high catecholamine response during intense exercise might be responsible for a certain dysregulation between glucose production and utilization. Whereas the glucose production increases 8-fold the glucose utilization increases only 3- to 4-fold, since catecholamines might impede glucose-dependent insulin secretion. [30] This recurrent hyperglycemia in combination with increasing age (accelerated hemoglobin glycation, prolonged erythrocyte turnover) could be responsible for rising HbA_1c_ proportions.

Taken this together, our data suggest different pathogeneses of dysglycemic states in elderly endurance athletes and age/sex matched controls. In controls, hypovitaminosis D_3_ correlates with high BMI and low physical performance, which can be together seen as surrogates for obesity. However, vitamin D_3_ is not only a surrogate of BMI, since 25(OH)D status presented as an independent predictor within the model, OR = 5.05, 95% CI (1.08, 23.63). Thus, low vitamin D_3_ levels might reflect a higher storage capacity of lipophilic compounds by increased adipose tissue that cannot be captured by mere BMI. In contrast to this, obesity was not a major issue in our studied marathoners/bicyclists. Moreover, BMI does not have the same value for estimating adiposity in athletes. Wallner-Liebmann and co-workers reported that male athletes showed a 50.3% lower total subcutaneous adipose tissue thickness when compared to male controls of the same BMI. As described above, hyperglycemic levels in athletes will be more likely reached via a catecholamine dependent discrepancy between glucose production and utilization, which leads to higher blood glucose levels during intense training and as a consequence to increased HbA_1c_ percentages.

Of course, this study comes with several limitations. First of all, the sample size of the study is moderate, nevertheless, the observed effect sizes were large enough in order to produce significant results. Indeed, the considerably small sample sizes are quite common in the field of marathon athletes: N = 125 [31], N = 18 [32], N = 15 [33]. This is mainly due to the limited size of the basic population of elderly marathoners. However, larger numbers would be needed if more subtle effects should be identified. Moreover, the proportion of female participants is very low, which is due to the lower number of female marathon runners. Although this number has been increasing during the last years, there are still less than two women among ten marathon finishers at the Vienna City Marathon. Amongst elderly the proportion of female participants is even smaller. Whereas female Austrian marathoners of any age group participating in the Vienna City Marathon 2015 had a share of 17.3%, this proportion shrunk to 4.7% amongst participants aged > 60 years.

To the best of our knowledge, this is the first manuscript that shows that the predictive value of lower 25(OH)D concentrations regarding the risk of (pre)diabetes might be influenced by intense exercise. In conclusion, our findings implicate that life style might modify the relevance of low 25(OH)D concentrations as a risk predictor of future morbidity in elderly patients.

## References

[1] Sattar N (2012) Biomarkers for diabetes prediction, pathogenesis or pharmacotherapy guidance? Past, present and future possibilities. Diabet Med 29: 5–13. 10.1111/j.1464-5491.2011.03480.x 21988593

[2] Poolsup N, Suksomboon N, Plordplong N (2015) Effect of vitamin D supplementation on insulin resistance and glycaemic control in prediabetes: a systematic review and meta-analysis. Diabet Med. 10.1111/dme.12893 26308752

[3] Wamberg L, Pedersen SB, Rejnmark L, Richelsen B (2015) Causes of Vitamin D Deficiency and Effect of Vitamin D Supplementation on Metabolic Complications in Obesity: a Review. Curr Obes Rep. 10.1007/s13679-015-0176-5 26353882

[4] Kabadi SM, Lee BK, Liu L (2012) Joint effects of obesity and vitamin D insufficiency on insulin resistance and type 2 diabetes: results from the NHANES 2001–2006. Diabetes Care 35: 2048–2054. 22751957 10.2337/dc12-0235 PMC3447853

[5] Clemente-Postigo M, Munoz-Garach A, Serrano M, Garrido-Sanchez L, Bernal-Lopez MR, et al (2015) Serum 25-hydroxyvitamin D and adipose tissue vitamin D receptor gene expression: relationship with obesity and type 2 diabetes. J Clin Endocrinol Metab 100: E591–595. 10.1210/jc.2014-3016 25706239

[6] Heidari B, Nargesi AA, Hafezi-Nejad N, Sheikhbahaei S, Pajouhi A, et al (2015) Assessment of serum 25-hydroxy vitamin D improves coronary heart disease risk stratification in patients with type 2 diabetes. Am Heart J 170: 573–579.e575. 10.1016/j.ahj.2015.06.017 26385042

[7] Nocon M, Hiemann T, Muller-Riemenschneider F, Thalau F, Roll S, et al (2008) Association of physical activity with all-cause and cardiovascular mortality: a systematic review and meta-analysis. European journal of cardiovascular prevention and rehabilitation: official journal of the European Society of Cardiology, Working Groups on Epidemiology & Prevention and Cardiac Rehabilitation and Exercise Physiology 15: 239–246. 10.1097/HJR.0b013e3282f55e09 18525377

[8] Gill JM, Cooper AR (2008) Physical activity and prevention of type 2 diabetes mellitus. Sports Med 38: 807–824. 18803434 10.2165/00007256-200838100-00002

[9] Sanchis-Gomar F, Lippi G (2014) Physical activity—an important preanalytical variable. Biochemia Medica 24: 68–79. 10.11613/BM.2014.009 24627716 PMC3936967

[10] Saraff V, Shaw N (2015) Sunshine and vitamin D. Arch Dis Child. 10.1136/archdischild-2014-307214 26323284

[11] Peeling P, Fulton SK, Binnie M, Goodman C (2013) Training environment and Vitamin D status in athletes. Int J Sports Med 34: 248–252. 10.1055/s-0032-1321894 22972245

[12] Haslacher H, Michlmayr M, Batmyagmar D, Perkmann T, Ponocny-Seliger E, et al (2015) Physical exercise counteracts genetic susceptibility to depression. Neuropsychobiology 71: 168–175. 10.1159/000381350 25998702

[13] Haslacher H, Michlmayr M, Batmyagmar D, Perkmann T, Ponocny-Seliger E, et al (2015) rs6295 [C]-Allele Protects Against Depressive Mood in Elderly Endurance Athletes. J Sport Exerc Psychol 37: 637–645. 10.1123/jsep.2015-0111 26866771

[14] Haslacher H, Perkmann T, Lukas I, Barth A, Ponocny-Seliger E, et al (2012) Myeloperoxidase levels predict executive function. Int J Sports Med 33: 1034–1038. 10.1055/s-0032-1304637 22855218

[15] Winker R, Lukas I, Perkmann T, Haslacher H, Ponocny E, et al (2010) Cognitive function in elderly marathon runners: cross-sectional data from the marathon trial (APSOEM). Wiener klinische Wochenschrift 122: 704–716. 10.1007/s00508-010-1485-z 21072603

[16] Böhm H, Bürklen R, Dienstl F, Ehrenböck G, Gaul W, et al (1978) Empfehlungen für eine standardisierte Ergometrie. Öst Ärzteztg 33: 333–344.

[17] American Diabetes A (2010) Diagnosis and Classification of Diabetes Mellitus. Diabetes Care 33: S62–S69. 10.2337/dc10-S062 20042775 PMC2797383

[18] Pearce SH, Cheetham TD (2010) Diagnosis and management of vitamin D deficiency. BMJ 340: b5664 10.1136/bmj.b5664 20064851

[19] Benjamini Y, Hochberg Y (1995) Controlling the false discovery rate: a practical and powerful approach to multiple testing. Journal of the Royal Statistical Society Series B (Methodological): 289–300.

[20] Hosmer DW, Lemeshow S, Sturdivant RX (2013) Applied Logistic Regression. Hoboken, NJ, USA: John Wiley & Sons, Inc.

[21] Lupton JR, Faridi KF, Martin SS, Sharma S, Kulkarni K, et al (2016) Deficient serum 25-hydroxyvitamin D is associated with an atherogenic lipid profile: The Very Large Database of Lipids (VLDL-3) study. J Clin Lipidol 10: 72–81.e71. 10.1016/j.jacl.2015.09.006 26892123 PMC4762185

[22] Zilinski JL, Contursi ME, Isaacs SK, Deluca JR, Lewis GD, et al (2015) Myocardial adaptations to recreational marathon training among middle-aged men. Circ Cardiovasc Imaging 8: e002487 10.1161/CIRCIMAGING.114.002487 25673646

[23] Nathan DM (2015) Diabetes: Advances in Diagnosis and Treatment. Jama 314: 1052–1062. 10.1001/jama.2015.9536 26348754

[24] Al-Shoumer KA, Al-Essa TM (2015) Is there a relationship between vitamin D with insulin resistance and diabetes mellitus? World J Diabetes 6: 1057–1064. 10.4239/wjd.v6.i8.1057 26240702 PMC4515445

[25] George PS, Pearson ER, Witham MD (2012) Effect of vitamin D supplementation on glycaemic control and insulin resistance: a systematic review and meta-analysis. Diabet Med 29: e142–150. 10.1111/j.1464-5491.2012.03672.x 22486204

[26] Song Y, Wang L, Pittas AG, Del Gobbo LC, Zhang C, et al (2013) Blood 25-Hydroxy Vitamin D Levels and Incident Type 2 Diabetes: A meta-analysis of prospective studies. Diabetes Care 36: 1422–1428. 10.2337/dc12-0962 23613602 PMC3631862

[27] Afzal S, Bojesen SE, Nordestgaard BG (2013) Low 25-hydroxyvitamin D and risk of type 2 diabetes: a prospective cohort study and metaanalysis. Clin Chem 59: 381–391. 10.1373/clinchem.2012.193003 23232064

[28] Wortsman J, Matsuoka LY, Chen TC, Lu Z, Holick MF (2000) Decreased bioavailability of vitamin D in obesity. Am J Clin Nutr 72: 690–693. 10966885 10.1093/ajcn/72.3.690

[29] Kratz A, Lewandrowski KB, Siegel AJ, Chun KY, Flood JG, et al (2002) Effect of Marathon Running on Hematologic and Biochemical Laboratory Parameters, Including Cardiac Markers. American Journal of Clinical Pathology 118: 856–863. 12472278 10.1309/14TY-2TDJ-1X0Y-1V6V

[30] Marliss EB, Vranic M (2002) Intense Exercise Has Unique Effects on Both Insulin Release and Its Roles in Glucoregulation: Implications for Diabetes. Diabetes 51: S271–S283. 11815492 10.2337/diabetes.51.2007.s271

[31] Hamstra-Wright KL, Coumbe-Lilley JE, Kim H, McFarland JA, Huxel Bliven KC (2013) The influence of training and mental skills preparation on injury incidence and performance in marathon runners. J Strength Cond Res 27: 2828–2835. 10.1519/JSC.0b013e31828a4733 23439344

[32] Agawa H, Yamada N, Enomoto Y, Suzuki H, Hosono A, et al (2008) Changes of mental stress biomarkers in ultramarathon. Int J Sports Med 29: 867–871. 10.1055/s-2008-1038490 18418810

[33] Morgan WP, Costill DL (1996) Selected psychological characteristics and health behaviors of aging marathon runners: a longitudinal study. Int J Sports Med 17: 305–312. 8814515 10.1055/s-2007-972852

